# Hydrogen, a potential safeguard for graft-*versus*-host disease and graft ischemia-reperfusion injury?

**DOI:** 10.6061/clinics/2016(09)10

**Published:** 2016-09

**Authors:** Lijuan Yuan, Jianliang Shen

**Affiliations:** I Anhui Medical University, Postgraduate School, Hefei, China; II Navy General Hospital, Department of Hematology, Beijing, China

**Keywords:** Molecular Hydrogen, Organ Transplantation, Graft-*Versus*-Host Disease, Ischemia-Reperfusion Injury, Antioxidant

## Abstract

Post-transplant complications such as graft-*versus*-host disease and graft ischemia-reperfusion injury are crucial challenges in transplantation. Hydrogen can act as a potential antioxidant, playing a preventive role against post-transplant complications in animal models of multiple organ transplantation. Herein, the authors review the current literature regarding the effects of hydrogen on graft ischemia-reperfusion injury and graft-*versus*-host disease. Existing data on the effects of hydrogen on ischemia-reperfusion injury related to organ transplantation are specifically reviewed and coupled with further suggestions for future work. The reviewed studies showed that hydrogen (inhaled or dissolved in saline) improved the outcomes of organ transplantation by decreasing oxidative stress and inflammation at both the transplanted organ and the systemic levels. In conclusion, a substantial body of experimental evidence suggests that hydrogen can significantly alleviate transplantation-related ischemia-reperfusion injury and have a therapeutic effect on graft-*versus*-host disease, mainly via inhibition of inflammatory cytokine secretion and reduction of oxidative stress through several underlying mechanisms. Further animal experiments and preliminary human clinical trials will lay the foundation for hydrogen use as a drug in the clinic.

## INTRODUCTION

Transplantation is a therapeutic modality in which healthy cells, tissues, or organs (an autograft or allograft) are transplanted to restore the anatomical structure and function of damaged organs or tissues. This approach is the final treatment choice for untreatable diseases and end-stage organ diseases 1. Organ transplantation leads to benefits such as functional recovery and prolonged survival, but post-transplantation complications such as ischemia-reperfusion (I/R) injury and acute graft-*versus*-host disease (aGVHD) remain major challenges 2-4. These complications reduce patients’ quality of life, increase medical costs, and worsen prognosis.

### Graft ischemia-reperfusion injury

During the process of transplantation, blood flow to the organ to be transplanted is interrupted, leading to ischemia that can damage the organ. In addition, restoration of blood flow to the transplanted organ may result in local and systemic inflammatory responses that can increase tissue injury. Graft I/R injury is characterized by reactive oxygen species (ROS) production, complement activation, leukocyte infiltration, platelet-leukocyte aggregation, increased microvascular permeability, and decreased endothelium-dependent relaxation 5,6. In the process of prolonged ischemia, adenosine triphosphate (ATP) levels and intracellular pH decrease because of anaerobic metabolism, leading to lactate accumulation. In addition, increased intracellular and mitochondrial calcium levels (calcium overload) are observed because certain ATPase-dependent ion transport mechanisms become dysfunctional 7. This calcium overload leads to cell swelling and rupture as well as cell death by necrotic, necroptotic, apoptotic and autophagic mechanisms.

Graft I/R injury is manifested by increased inflammation mediated by the complement system and cytokines. Once activated, the complement pathway damages the transplanted organ’s cells by attacking the plasma membrane or recruiting/activating neutrophils 8,9. Cytokines may play either pro- or anti-inflammatory roles. Among others, tumor necrosis factor (TNF)-α is central in graft I/R injury 10. Increased TNF-α in the graft will lead to increased neutrophil recruitment, increased ROS production and activation of the NF-κB and JNK pathways 11. Other cytokines are also involved: interleukin (IL)-1β, IL-18, and interferon (IFN)-γ increase damage, while IL-6, IL-10, and IL-13 attempt to control the damage 10. In its severest form, I/R injury can lead to dysfunction and possibly death of the transplanted organ 5,6,12.

### Graft-*versus*-host disease

GVHD is a severe complication of organ transplantation, resulting in morbidity and mortality. GVHD consists of three phases, and several of the involved mechanisms are shared with the classical mechanisms of I/R injury. During the first phase, tissues are damaged by the recipient’s conditioning regimen, which leads to the release of inflammatory cytokines such as TNF-α, IL-1, and IL-7 13. These cytokines induce activation of host antigen-presenting cells (APCs). During the second phase of GVHD, the host APCs activate the donors’ cells through IL-12 and IL-23 release, resulting in the production of Th1-related cytokines such as IL-2, IL-6, and TNF-γ. IL-10 downregulates the synthesis of these cytokines, but it is usually itself downregulated in the inflammatory context. The activated Th1 cells from the donor secrete IFN-γ to induce secretion of indoleamine 2.3-dioxygenase by the host APCs, thus stimulating immunotolerizing Tregs. IFN-γ also stimulates mononuclear cells to secrete IL-1 and TNF-α, which are inflammatory cytokines 13. Finally, in the third phase, the Th1 cells promote the proliferation and differentiation of cytotoxic T lymphocytes (CTLs) and stimulate natural killer (NK) cells, which in turn induce apoptosis of the cells of the transplanted organ 14. Cellular and inflammatory cytokines such as TNF-γ, IL-1, and IL-6 then directly assault various host tissues, leading to the clinical manifestations of GVHD 15. The activated cells also produce ROS, resulting in severe cell damage and the development of GVHD 16. Several studies have shown that GVHD is characterized by increased oxidative stress 17-19, and it has thus been suggested that antioxidants could be used to prevent GVHD 20.

### Hydrogen as an antioxidant

Hydrogen is an inert gas that was long considered to have no effect on higher living organisms. Interestingly, however, in 2007, Ohsawa et al. 21 observed that hydrogen could reduce the levels of hydroxyl radicals (the most cytotoxic of all ROS), effectively protecting cells 22. Subsequently, many studies showed that hydrogen acts as an antioxidant and it has been used broadly in the prevention and treatment of many illnesses in experimental animal models 23-28. The hydrogen used in these studies mainly consisted of two types (hydrogen gas and hydrogen-rich saline) delivered through a number of methods, such as ventilation with mixed gas containing hydrogen 29, oral administration of hydrogen-rich saline 30, intraperitoneal injection of hydrogen gas or hydrogen-rich saline 31, and intravenous injection of hydrogen-rich saline 32.

### Hydrogen as a therapeutic modality against transplantation-related I/R injury and GVHD

Increasing evidence has shown that molecular hydrogen could play an important role in the prevention and treatment of GVHD and graft I/R injury. Hence, the current literature regarding the effects of hydrogen on different animal models mimicking GVHD and I/R injury will be reviewed. The mechanisms of hydrogen’s effects on I/R injury and GVHD are summarized in Table 1, but although many studies were performed in models of I/R injury, these were not necessarily models of graft I/R injury. Therefore, the results may provide clues about the use of hydrogen for the treatment of graft I/R injury, but caution must be taken when examining these results. The studies mainly examined the use of hydrogen for organ pre-conditioning before harvesting, during organ preservation, and just before or during transplantation. Indeed, prolonged hypothermic preservation prior to transplantation is a challenge in the process of transplantation. In addition, graft I/R injury is common during transplantation, wherein multiple factors are involved and contribute to ROS production and ultrastructural injury. A number of studies have shown that hydrogen can decrease inflammation and apoptosis in graft organs, as detailed below.

### Heart

Nakao et al. 33 showed that hydrogen could significantly reduce heart I/R injury induced by prolonged hypothermic preservation prior to transplantation through hydrogen’s anti-inflammatory and antioxidant properties, as revealed by decreased levels of malondialdehyde (MDA) (an oxidation marker) and levels of troponin I and creatine phosphokinase (markers of heart injury). Similarly, Noda et al. 34 documented that a novel hydrogen-supplemented preservation solution efficiently improved myocardial injury due to cold I/R in a rat heterotopic transplantation model. In this study, the hydrogen-rich preservation solution led to decreased levels of IL-6, IL-1β, TNF-α, ICAM-1, iNOS, and CCL2, which are all markers of inflammation. Another study by Noda et al. showed that drinking hydrogen-rich water after heart transplantation could enhance cardiac allograft survival due to hydrogen’s antioxidant properties, eliminating toxic ROS and reducing chronic intimal hyperplasia of the aortic artery after heart and artery transplantation 35. Indeed, this study showed that hydrogen led to increased ATP levels and more efficacious mitochondrial respiratory chain function as well as decreased IL-2 and IFN-γ levels and intimal hyperplasia 35. Therefore, the use of hydrogen in heart transplantation seems to be associated with reduced oxidative stress and inflammation. However, the available studies on heart grafts are limited in number and scope.

### Kidneys

Hydrogen-rich saline from the University of Wisconsin has been used during hypothermic preservation of renal grafts and has been shown to decrease oxidative stress, as represented by lower MDA and serum 8-hydroxydeoxyguanosine (8-OHdG) levels as well as by prolonged graft survival 36. This preservation solution also decreased macrophage infiltration of this type of graft 36. In animal models of kidney transplantation, Shingu et al. 37 showed that treatment with hydrogen-rich saline could significantly attenuate renal graft I/R injury by reducing the levels of 8-OHdG, therefore improving renal transplant function and maintaining normal tissue structure after transplantation. Cardinal et al. 38 found that oral administration of hydrogen-rich saline could improve kidney function and increase overall survival after allotransplantation through reduction of oxidative stress and limited activation of inflammatory pathways such as MAPK pathways. Taken together, these results suggest that hydrogen improves kidney graft outcomes by decreasing inflammation and oxidative stress.

### Lungs

One study showed that lung inflation with 3% hydrogen during the cold ischemia phase lowered graft myeloperoxidase (MPO) activity and serum IL-8 and TNF-α levels, resulting in alleviated lung graft injury and improved function 39. A study by Noda et al. 40 showed similar results in a rat model of lung transplantation using hydrogen preconditioning during the *ex vivo* period. The study also showed that the levels of hypoxia-inducible factor-1 were decreased in hydrogen-treated lungs, leading to decreased levels of the inflammatory cytokines IL-6, IL-1β, and TNF-α 40. In rat models of brain-dead donor/recipient lung transplantation, it was demonstrated that hydrogen inhalation by the donors and the recipients could improve both lung function and graft histology by decreasing the inflammatory index (higher IL-8 and lower TNF-α levels), oxidative stress (increased superoxide dismutase (SOD) activity and lower MDA levels), and apoptosis 41. In another rat lung transplantation model, inhalation of mixed gas (98% oxygen and 2% hydrogen) alleviated lung graft I/R injury 42 by reducing inflammatory mediator upregulation as well as macrophage infiltration and sequestration; lowering tissue MDA levels 2 hours after reperfusion; and increasing the levels of the B-cell lymphoma (Bcl)-2 and Bcl-extra-large proteins, two proteins involved in apoptosis. A study by the same group showed that inhalation of mixed gas (2% hydrogen and 98% oxygen) by the organ donor could reduce the severity of I/R injury by reducing tissue edema and the number of apoptotic pulmonary epithelial cells and especially by increasing the expression of heme oxygenase-1 (HO-1), which is a potent, inducible transcription factor with antioxidant, anti-inflammatory, and anti-apoptotic properties, therefore playing important roles in lung graft protection 43. Additionally, a recent study in pigs showed that hydrogen gas inhalation during *ex vivo* lung perfusion improved lung function after donation following cardiac death; the hydrogen group also had lower expression of IL-1β, IL-6, IL-8, and TNF-α as well as lower scores for lung injury severity 44. Taken together, these studies all suggest that the use of hydrogen in lung grafts reduces inflammation, oxidative stress, and apoptosis.

### Liver

One study examined the outcomes of perfusing the donor liver with hydrogen-saturated lactate Ringer’s solution just before reperfusion and showed significantly lower aspartate aminotransferase and lactate dehydrogenase levels in animals with hydrogen-perfused livers, suggesting better graft function than in untreated grafts 45. In a rat model of small intestinal transplantation wherein both donors and recipients received 2% hydrogen inhalation, Buchholz et al. 46 found that hydrogen treatment significantly decreased the levels of CCL2, IL-1β, IL-6, and TNF-α, leading to improved gastrointestinal transit and decreased lipid peroxidation as well as attenuated post-transplant breakdown of mucosal barrier function. Shigeta et al. 47 showed that luminal injection of hydrogen-rich solution attenuated I/R injury in a rat model of intestine transplantation by reducing oxidative stress. In a rat model of pancreas transplantation, hydrogen-rich saline was shown to protect against I/R injury, as demonstrated by better histopathological damage scores (based on edema, inflammation and necrosis) and better pancreatic function as well as by reduced levels of TNF-α, IL-1β, and IL-6 48. In a rat model of small-bowel transplantation, rats suffered from symptoms of gastroparesis, and Buchholz et al. 49 showed that hydrogen could alleviate this transplantation-related gastroparesis by improving the suppression of graft muscle contractility, inhibiting the gene expression of proinflammatory factors, and reducing the systemic inflammatory response.

### Bone marrow and GVHD

Few studies have specifically examined the effects of hydrogen on GVHD. Qian et al. 50 studied the effects of hydrogen-rich saline treatment in a mouse model of haploidentical allogenic bone marrow transplantation and found that the hydrogen-rich saline group had significantly reduced GVHD, significantly higher survival and faster recovery of peripheral blood leukocytes compared with the control group. This study further expanded the application range of hydrogen and introduced a new method for treating GVHD. However, the study did not examine the exact mechanisms involved in the results and suggested that reduced TNF-α, IL-2, and/or ROS levels may play roles in the benefits of hydrogen against aGVHD after bone marrow transplantation 50. In a mouse model consisting of lethal irradiation followed by allogeneic hematopoietic stem cell transplantation, hydrogen-rich saline was shown to improve the survival rate, to lower the rate of GVHD and the serum levels of inflammatory cytokines, and to reduce tissue damage 51. Again, the exact mechanisms involved have not been explored.

### Hydrogen as a treatment for non-graft I/R injury

A number of studies have shown that hydrogen could be used to prevent I/R injury. In an experimental model of bilateral renal pedicle occlusion for 45 minutes, Wang et al. 31 showed that intraperitoneal injection of hydrogen-rich saline five minutes before reperfusion could alleviate I/R injury by inhibiting the secretion of a variety of inflammatory cytokines. In a rat model of I/R injury, inhalation of 2.5% hydrogen initiated 10 minutes before reperfusion and continued for 120 minutes could attenuated renal I/R injury by decreasing MDA levels 52. Sun et al. 53,54 also showed that hydrogen could reduce myocardial damage in a rat heart with regional myocardial I/R through antioxidative and anti-inflammatory effects.

In a New Zealand white rabbit model of lung I/R injury, hydrogen-rich saline treatment protected the lung from I/R injury by increasing the PaO_2_/FiO_2_ ratio and reducing the lung wet/dry ratio 55; in particular, the hydrogen-rich saline group displayed a significantly lower proportion of alveolar hemorrhage and pathologic lesions compared with the control group. In a rat model of lung injury induced by intestinal I/R injury, Mao et al. 56 showed that hydrogen-rich saline could reduce lung injury by decreasing MDA levels and MPO activity in the lung tissues.

In a liver injury mouse model, Sun et al. 57 showed that hydrogen-rich saline treatment could have protective effects on the liver. Similarly, in a mouse model of liver I/R injury, Fukuda et al. 58 found that hydrogen inhalation could significantly reduce liver I/R injury by inhibiting the release of serum alanine aminotransferase and MDA production.

Spinal cord injuries can be divided into two phases: i.e., direct mechanical tissue disruption, followed by cell damage by a cascade that includes oxidative stress, calcium mobilization, glutamate toxicity, and inflammation 59. Increased ROS production during spinal cord injuries plays a role in neuronal death and subsequent neuronal deficits 60,61. In addition to causing direct insults to macromolecules, these ROS act as intracellular messengers of neuronal death 62,63. Neurons are among the cells most sensitive to ROS 59. It was found that hydrogen reduced acute spinal cord contusion injury by increasing the release of brain-derived neurotrophic factor and decreasing the levels of oxidative products such as 8-iso-prostaglandin F2α and MDA 64,65.

Retinal I/R injuries are often observed in conditions such as acute angle-closure glaucoma, retinal artery occlusion, and amaurosis fugax. In animal models, retinal I/R injuries are often induced by transient elevation of intraocular pressure. In a model of retinal I/R injury, hydroxyl radicals caused irreversible cellular damage by affecting lipids, proteins and nucleic acids 66. Hydrogen-loaded eye drops were used in these animals and markers such as 4-hydroxynonenal and 8-hydroxy-2-deoxyguanosine were used to evaluate I/R injury. The hydrogen-loaded eye drops dramatically decreased 4-hydroxynonenal and 8-hydroxy-2-deoxyguanosine levels and reduced subsequent retinal cell death after I/R injury 66.

Testicular torsion occurs when the spermatic cord twists, thereby cutting off the testicle’s blood supply. This urological condition usually affects children and adolescents and inflammatory cytokines and free radicals play important roles. One study assessed the effect of hydrogen-rich saline on testicular I/R injury after testicular torsion and showed that the injury score in the hydrogen treatment group was the lowest among all tested groups. Moreover, compared with the other groups, in the hydrogen treatment group, MDA levels were significantly lowered and SOD activity was significantly improved 67.

In a rat model of *in utero* I/R injury, Mano et al. 68 studied the effects of hydrogen on rat fetal hippocampal damage caused by I/R on day 16 of pregnancy. The results indicated that oral administration of hydrogen-saturated water could reduce placental oxidative damage, alleviate neonatal growth retardation and improve the rat fetal hippocampal damage caused by *in utero* I/R 68. Similar effects were observed in another study 69.

Therefore, a number of animal experiments over the last few years have shown that hydrogen can obviously reduce the damage caused by organ transplantation. From the initial simple effect of antioxidative activity 23 to anti-inflammatory and anti-apoptotic activity and regulation of signaling pathways 31,54,64,70, the effects of hydrogen treatment have been demonstrated in many experiments.

A substantial body of experimental evidence suggests that hydrogen can significantly alleviate I/R injury related to transplantation and has a therapeutic effect on complications of transplantation (including GVHD), mainly *via* inhibition of inflammatory cytokine secretion and reduction of oxidative stress. However, the exact mechanisms leading to these effects are currently ill known. In addition, many studies on the effects of hydrogen were performed in models of I/R injury, but not in models of graft I/R injury. Nevertheless, the results may provide clues about the use of hydrogen for the treatment of graft I/R injury, although caution must be taken when examining these results.

With the advantages of being easily available, having a low price, and being a nontoxic small molecule that is easily absorbed, hydrogen has a strong prospect of clinical applications. Further animal experiments and preliminary human clinical trials are needed to lay the foundation for hydrogen use as a drug in the clinic in the near future.

## AUTHOR CONTRIBUTIONS

All authors contributed to the review of the literature, the data analysis, and the manuscript writing and approved the final version of the manuscript.

## Figures and Tables

**Table 1:** Mechanisms of the therapeutic effects of hydrogen on ischemia-reperfusion injury and graft-*versus*-host disease.

Effects	Mechanism
**Antioxidation**	*Inhibition of increased myeloperoxidase (MPO) activity 56
	*Elimination of toxic reactive oxygen species 35,46
	*Decreased levels of 8-hydroxydeoxyguanosine 36,37,66
	*Decreased tissue malondialdehyde levels 36,42,52,56,64,65,67
	*Decreased lipid peroxidation 42,49
	*Increased expression of heme oxygenase-1 43
	*Decreased MPO activity 56
	*Decreased levels of 8-iso-prostaglandin F2α 64,65
	*Decreased levels of 4-hydroxynonenal 66
	*Improved superoxide dismutase activity 67
	*Decreased hypoxia-inducible factor-1 levels 40
**Anti-inflammation**	*Inhibition of the secretion of a variety of inflammatory cytokines 31,42,46,56
	*Decreased inflammatory index and oxidative stress 41
	*Reduced macrophage infiltration and sequestration 42
	*Reduced recruitment of neutrophils 46
	*Inhibition of the gene expression of proinflammatory factors 49
**Anti-apoptosis**	*Increased levels of the B-cell lymphoma-2 and Bcl-extra-large proteins 42
	*Reduced number of NF-κB-positive cells 71
**Other**	*Inhibition of the release of serum alanine aminotransferase 58
	*Improved suppression of the graft muscle contractility induced by transplantation 49
	*Increased release of brain-derived neurotrophic factor 64,65
	*Regulation of signaling pathways 54,64,70,71
